# The human miRNA repertoire of different blood compounds

**DOI:** 10.1186/1471-2164-15-474

**Published:** 2014-06-14

**Authors:** Petra Leidinger, Christina Backes, Benjamin Meder, Eckart Meese, Andreas Keller

**Affiliations:** Institute of Human Genetics, Saarland University, Homburg, Germany; Chair for Clinical Bioinformatics, Saarland University, Saarbrücken, Germany; Internal Medicine III, University Hospital Heidelberg, Heidelberg, Germany

**Keywords:** miRNA, Exosome, Cell separation

## Abstract

**Background:**

MiRNAs from body fluids gain more and more attraction as biomarker candidates. Besides serum, patterns from whole blood are increasingly considered as markers for human pathologies. Usually, the contribution of different cell types to the respective signature remains however unknown. In this study we provide insights into the human miRNome of different compounds of the blood including CD3, CD14, CD15, CD19, CD56 positive cells as well as exosomes.

**Methods:**

We measured the miRNA repertoire for each cell type and whole blood for two individuals at three time points over the course of one year in order to provide evidence that the cell type miRNomes can be reproducibly detected.

**Results:**

For measurements repeated after 24 hours we found on average correlation of 0.97, even after one year profiles still correlated with 0.96, demonstrating the enormous stability of the cell type specific miRNomes. Highest correlation was found for CD15 positive cells, exceeding Pearson correlation of 0.99. For exosomes a significantly higher variability of miRNA expression was detected. In order to estimate the complexity and variability of the cell type specific miRNomes, we generated profiles for all considered cell types in a total of seven unaffected individuals. While CD15 positive cells showed the most complex miRNome consisting of 328 miRNAs, we detected significantly less miRNAs (186, p = 1.5*10^-5^) in CD19 positive cells. Moreover, our analysis showed functional enrichment in many relevant categories such as onco-miRNAs and tumor miRNA suppressors. Interestingly, exosomes were enriched just for onco-miRNAs but not for miRNA tumor suppressors.

**Conclusion:**

In sum, our results provide evidence that blood cell type specific miRNomes are very consistent between individuals and over time.

## Background

Small non-coding RNAs (miRNAs) are involved in the majority of biological processes, including proliferation, apoptosis, differentiation, and development [1–3]. Moreover, the majority of human genes are regulated by miRNAs. Not surprisingly, miRNAs also play a crucial role in pathogenic processes.

In the beginning of miRNA research mostly tissue has been in the focus of research, thus for all human tissue types specific profiles have been discovered, e.g. from patients with lung cancer [4], breast cancer [5] or glioblastoma [6]. More recently body fluids as source for non-invasive or minimal-invasive markers have become more important. Besides serum (biomarker discovery for non-ischaemic systolic heart failure [7], pulmonary tuberculosis [8], non small-cell lung cancer [9, 10], breast cancer [11], prostate cancer [12], or ovarian cancer [13]), one of the most frequently applied approaches remains to measure miRNAs from whole blood (biomarkers for myocardial infarction [14], lung cancer [15], multiple sclerosis [16, 17], melanoma [18], ovarian cancer [19], COPD [20], glioblastoma [21] and Alzheimer’s Disease [22]). As miRNAs are known to be very specific for different tissues, they are likewise known to be specific for different compounds of the blood. The respective knowledge on the origin of blood-borne miRNA patterns has however been hardly considered.

In our study we aimed to provide miRNomes for common compounds of the human blood, including CD3, CD14, CD15, CD19, CD56 positive cells and exosomes. For all samples we also provided the measurement of whole blood using PAXgene tubes as comparison. In a first stage, we measured the miRNA repertoire for each cell type and whole blood for two individuals at three time points (t0; t1 = t0 + 1 year; t2 = t1 + 1 day) in order to get an estimation how stable cell type specific miRNomes are over time. Here, we detected a very high reproducibility and stability for all tested cell types.

In a second stage we extended the measurement to 7 individuals in order to understand the complexity and variability of cell type specific miRNomes. Our results showed that the cell type miRNomes are highly specific but comparably constant between different individuals. Thus, our study represents a standard repertoire of miRNAs in the considered cell types.

## Results and discussion

### Stability of the human blood compound miRNomes

A central question of our study was how reproducible and reliable can miRNomes of individuals be measured. To this end we picked one male and one female individual and collected blood at three time points: t0, t1, which was one year after t0 and t2, which was one day after t1. Blood cell type extraction and detection of the respective miRNomes was done immediately after blood collection. This set-up allows us to test for the reproducibility of cell type specific miRNomes over a very short period of time but at the same time offers to report the stability of the miRNA repertoire in individuals over a longer period.

For measurements repeated within 24 hours we found on average a high correlation of 0.97, indicating a reasonable reproducibility of the blood collection, cell type separation and miRNA profiling approach. Astonishingly, profiles correlated even after one year still very well. On average, Pearson correlation was as high as 0.96, demonstrating the enormous stability of the cell type specific miRNomes over a longer period of time.

**Figure 1:**
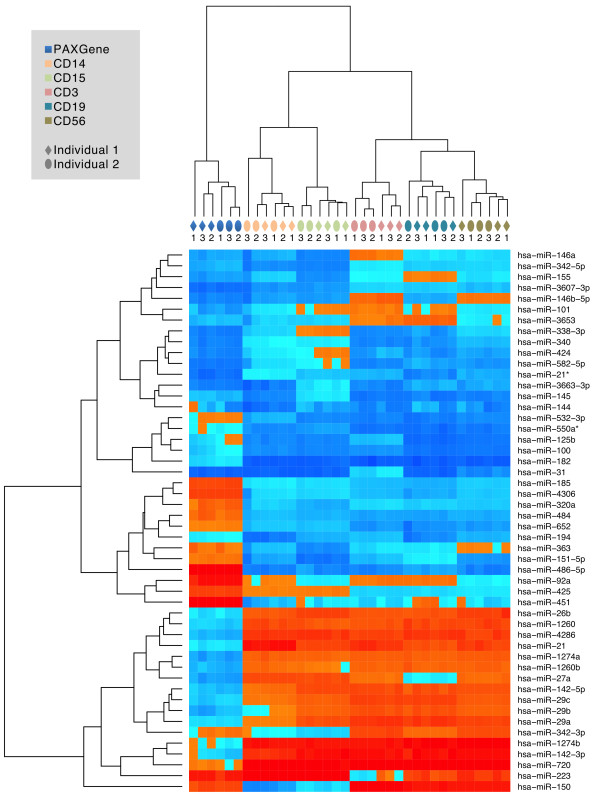
**Cluster Heatmap.** The figure presents clustering in different blood cell types (represented by different colors on top of the heatmap) for two individuals (represented by two different shapes on top of heatmap) at three different timepoints (represented by numbers on top of heatmap).

Considering the two consecutive time points, the highest correlation for a specific cell type was reached by CD15 positive cells, exceeding 0.99. Just CD3 positive cells reached almost the same level of reproducibility. A similar behavior was also observed for the correlation within cell types in general. Here, CD3, CD15 and CD56 positive cells reached correlation of 0.98, CD14 positive cells and PAXgene whole blood reached average correlation of 0.97, CD19 positive cells showed lowest but still substantial correlation of 0.96.To understand how the different cell populations are related to each other we applied hierarchical clustering on the 50 most variable miRNAs. As the dendrogram in Figure 1 presents, different cell types cluster perfectly to each other. PAXgene blood shows the most different patterns, CD14 and CD15 positive cells clustered well together as well as CD3, CD19 and CD56 positive cells. Notably, for PAXgene blood and CD3 positive cells, individuals likewise cluster together. Regarding the three time points we just observed a tendency of the latter two time points to cluster well together, again demonstrating the high stability of blood cell type specific miRNomes over a longer period of time.

### Complexity and specificity of cell type specific miRNomes

**Figure 2:**
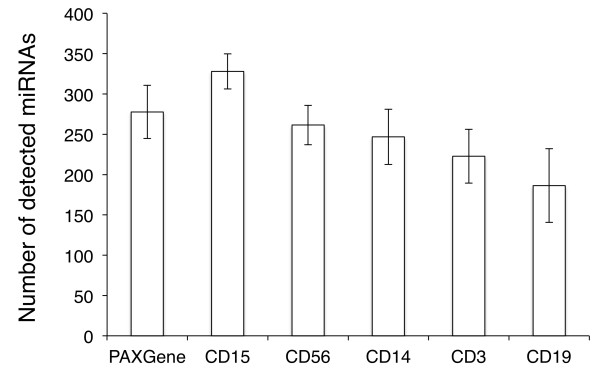
**Bar diagram detaining the number of different miRNAs in all tested cell compounds.**

**Figure 3:**
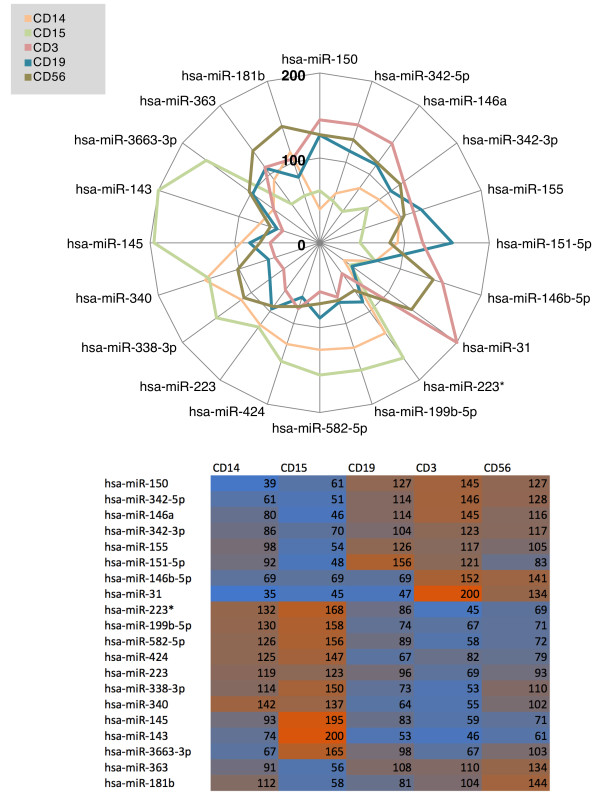
**Spider diagram showing the abundance of selected miRNAs in different cell types.** Total blood cells have been used to normalize the data (100%).

In order to improve our understanding of the complexity and specificity of cell type miRNomes we extended our cohort to 7 unaffected individuals and measured the same cell fractions. The bar diagram in Figure 2 presents the average number of detected miRNAs per cell fraction. While CD15 positive cells showed the most complex miRNome consisting of as much as 328 of the measured 1,205 markers, we detected significantly less miRNAs (186, p = 1.5*10^-5^) in CD19 positive cells. CD56, CD14 and CD3 positive cells showed comparable number of miRNAs with 262, 247 and 223 detected miRNAs on average. The respective miRNomes per cell fraction are available in Supplemental Table 1.Based on the different complexity in miRNome we concluded that the respective profiles as such may also show a high degree of variability. To validate this hypothesis we focused on the miRNAs with highest variance among all cell types and calculated the expression as percentage of average expression over all cell types for each miRNA. As the spider diagram and heatmap in Figure 3 detail, we discovered 6 miRNAs being significantly higher expressed in CD19, CD3 and CD56 positive cells as compared to CD14 and CD15 positive cells: miR-150, miR-342-5p, miR-146a, miR-342-3p, miR-155 and miR-151-5p. 7 miRNAs showed the opposite behavior: miR-223*, miR-199b-5p, miR-582-5p, miR-424, miR-223, miR-338-3p and miR-340. These results are in line with our initial findings on the two individuals measured over 1 year, overall CD14 and CD15 positive cells have similar patterns as well as CD19, CD3 and CD56 positive cells. However, we also detect miRNAs that are highly expressed just in single cell types, such as miR-145, miR-143 and miR-3663-3p for CD15 positive cells. Vice versa, miR-363 and miR-181b are over expressed in CD56 positive cells and show significantly decreased expression in CD15 positive cells.

**Table 1:** **Enriched clusters, families and functions**

	CD14	CD15	CD19	CD3	CD56	EXOSOMES
**hsa-mir-106a cluster**	0,0281	0,0903	0,0676	0,1882	0,0576	0,7784
**hsa-mir-106b cluster**	0,0932	0,181	0,0377	0,0915	0,1382	0,2424
**hsa-mir-17 cluster**	1,74E-03	8,98E-03	7,50E-03	0,0294	5,03E-03	0,1644
**hsa-mir-181c cluster**	0,3601	0,025	0,1888	0,0874	0,0151	0,1233
**let-7 family**	8,20E-04	4,67E-04	8,46E-05	4,66E-05	1,68E-04	1
**mir-15 family**	0,2459	0,0701	8,05E-03	0,0288	0,0498	0,0704
**mir-29 family**	0,0963	0,1906	0,0393	0,0944	0,1441	NA
**mir-30 family**	0,0917	0,026	0,0308	0,09	0,1462	1
**mir-320 family**	0,0267	0,0736	8,16E-03	0,0291	0,0512	6,88E-03
**Akt pathway**	5,97E-04	8,81E-03	7,17E-03	3,90E-03	3,43E-03	1
**Angiogenesis**	2,38E-05	6,49E-06	7,32E-05	5,62E-04	3,56E-05	5,11E-03
**Apoptosis**	2,32E-05	1,06E-04	1,26E-07	1,39E-06	1,11E-05	0,1011
**Bone regeneration**	4,25E-03	0,0488	0,1377	0,0465	0,0141	1
**Cell cycle related**	8,25E-04	1,18E-03	1,76E-05	1,31E-05	6,85E-04	0,0214
**Cell proliferation**	0,0142	9,84E-03	5,48E-04	5,20E-03	0,029	0,1563
**Chemosensitivity of tumor cells**	0,0262	0,068	7,86E-03	0,0282	0,0486	0,0672
**Folliculogenesis**	0,0741	0,0386	0,0192	9,54E-03	0,1328	0,2249
**Granulopoiesis**	2,85E-04	1,66E-04	1,96E-05	2,48E-04	9,81E-04	0,1486
**HCV infection**	0,5524	0,8972	0,2993	0,5554	0,7399	0,0363
**HIV latency**	1,69E-03	8,77E-03	2,27E-03	8,77E-03	0,0102	0,058
**Hormones regulation**	7,30E-09	9,82E-07	9,80E-09	3,25E-08	1,52E-08	4,69E-03
**Human embryonic stem cell regulation**	0,0379	8,71E-03	0,0312	5,54E-03	6,97E-03	0,0664
**Immune response**	2,40E-05	1,57E-05	6,88E-07	5,56E-06	4,32E-06	0,0105
**Inflammation**	0,23	0,0413	0,037	0,0563	0,0595	0,6727
**miRNA tumor suppressors**	4,66E-04	1,18E-06	1,76E-05	1,85E-06	1,04E-05	0,1986
**onco-miRNAs**	4,49E-06	9,76E-05	7,84E-08	3,34E-06	2,83E-06	6,08E-03

**Figure 4:**
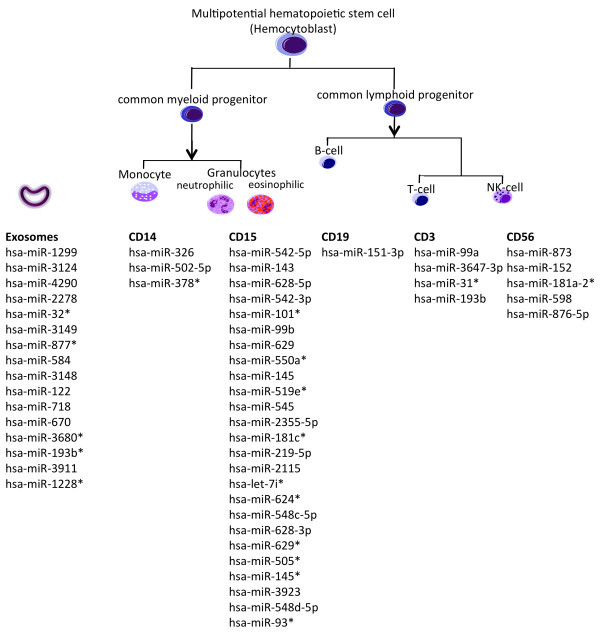
**miRNAs that are specific for the different cell compounds.** The miRNAs below the respective compounds have been detected just in the respective compounds while not being available in any other type.

Notably, we discovered for each cell fraction at least a single specific miRNA, i.e. a miRNA that was just expressed in this cell type (in two experiments) while not in any other cell type. For CD56 positive cells highest number of specific miRNAs was detected (25), in contrast, the other fractions showed substantially less specific markers: CD14 (3), CD56 (5), CD3 (4) CD19 (1). The respective specific markers are summarized in Figure 4. The full miRNome profile of the respective individuals for all cell types is available in Supplemental Table 2.

**Table 2:** **Information for all blood donors**

Blood donor	Gender	Age	Health status
**h1**	female	32	healthy
**h2**	female	45	bee venom allergy
**h3**	male	55	pollen allergy
**h4**	female	31	healthy
**h5**	male	39	healthy
**h6**	female	49	healthy
**h7**	female	48	Von Hippel-Lindau disease

### The miRNome profile of exosomes

Besides measuring whole miRNome profiles from different tissues or body fluids like whole blood, serum or urine, miRNA profiles of exosomes that are released by cells and circulate in body fluids become likewise more central for current research. Thus, we also extracted exosomes from serum of the same two individuals at t1 and t2 and measured the respective exosome miRNomes. Repeated extraction of exosomes of the same individuals correlated well to each other, on average, pearson correlation was above 0.95. While this still represents a reasonable stability of exosome profiles, the values were clearly below measurements for the different cell types, corresponding to a higher variability of miRNAs in exosomes.Altogether, we found just a small number of miRNAs being expressed in these microvesicles. On average, 128 miRNAs were detected, a number well below the average number of detected miRNAs in the different analyzed blood cell types from the same blood donors. Despite the lower complexity of exosome miRNA profiles we searched for miRNAs being present just in exosomes while not in any other cell fraction. Indeed, we detected markers present at least in two different exosome measurements while not in any blood cell type or whole blood of the same individuals. The respective 16 markers include hsa-miR-1299, hsa-miR-3124, hsa-miR-4290, hsa-miR-2278, hsa-miR-32*, hsa-miR-3149, hsa-miR-877*, hsa-miR-584, hsa-miR-3148, hsa-miR-122, hsa-miR-718, hsa-miR-670, hsa-miR-3680*, hsa-miR-193b*, hsa-miR-3911 and hsa-miR-1228* (see Figure 4).

### Family and function of blood cell compound miRNAs

In order to find potentially enriched miRNA families or clusters as well as biological functions we used the freely available TAM tool [23]. Specifically, we analyzed all miRNAs for each blood compound that has been detected at least in 2 measurements. Our analysis revealed that in many cases specific miRNA families are enriched in selected cell types, for CD14 positive cells, miR-106a cluster was significant while for CD19 positive cells miR-106b cluster was found to be enriched. Interestingly, miR-17 cluster was significant for all cell types but not for exosomes. The same holds for the Akt pathway, Cell proliferation, Apoptosis, Granulopoiesis and others. Most interestingly, miRNA tumor suppressors were enriched in all categories besides exomes, however, in the case of onco-miRNAs, exosomes became likewise significant. All results are summarized in Table 1.

## Conclusion

While for gene expression various studies have been carried out in order to systematically explore patterns in different cell types, for miRNAs few studies have been published [24, 25]. As an example, Yu and co-workers demonstrated that positive and negative selection do not have a significant influence of the miRNA pattern [26]. In order to reduce the risk of contamination with other cells and to reach high purity of the isolated cell fractions we decided to apply positive cell selection. In two stages we then systematically explored the specific miRNomes of 5 cell fractions including CD3, CD14, CD15, CD19 and CD56. We also performed profiling of the same individuals using PAXgene blood tubes, representing one of the gold standard approaches for detecting circulating disease markers for various human pathologies. Even more importantly, we likewise generated profiles from exosomes of the respective individuals.

In summary our study reveals that 1) miRNA patterns of specific compounds of human blood can be reliably measured and are stable even over the course of one year; 2) the different cell types show substantial differences with respect to the complexity of their respective miRNomes; 3) CD14 and CD15 positive cells have a common myeloid progenitor and show a similar expression pattern, likewise CD3, CD19 and CD56 positive cells (common lymphoid progenitor) are generally related to each other; 4) Exosomes show a less complex and more variable miRNA profile as compared to other cell types.

## Methods

### Study set-up and miRNA profiling

**Figure 5:**
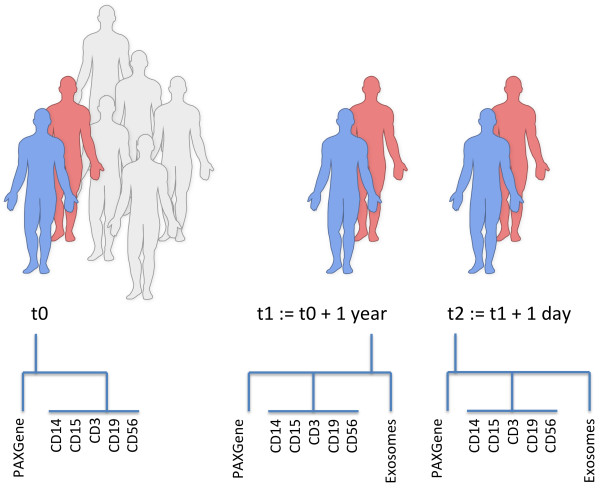
**Study set-up.** The diagram shows the different time points and cohort sizes for which the blood cell compounds were calculated.

The study set-up is presented in Figure 5. All participants in the study gave written informed consent. The local ethics committee (Ärztekammer des Saarlandes) approved the study (reference: 01/08). From all blood donors (details see Table 2), blood was collected in PAXgene RNA blood tubes (Becton Dickinson) and in S-Monovette® with EDTA K2 (Sarstedt). RNA from PAXgene whole blood was extracted according to manufacturers instruction using the PAXgene Blood miRNA Kit (Qiagen). For different cell types, positive selection was applied. While for CD3, CD19, CD15, and CD14 positive cells non-magnetic beads along with a sieve (pluriSelect) were used, for CD56 positive cells magnetic beads (Miltenyi Biotech) were used. The selection has been carried out using the leukocyte fraction. Exosome extraction has been carried out using ExoQuick Exosome Precipitation Solution (System Biosciences). Total RNA including small RNAs was isolated from different cell types and from exosomes using miRNeasy Micro Kit (Qiagen) according to manufacturer’s instructions.

Expression analysis was performed as previously described using Agilent’s Sure Print G3 Human v16 8x60K miRNA microarrays [27]. These arrays contain 40 replicates of each of the 1,205 miRNAs as annotated in miRBase v16 [28].

### Bioinformatics analysis

The initial data analysis was performed using Agilent’s Feature Extraction software as described in the manufacturers instructions. For further down-stream analysis, the 40 replicates of background corrected miRNA measurements were summarized using Agilent’s Feature Extraction software. These measurements are available as raw data at GEO with the reference GSE56590. The further down-stream analysis has been carried out using the freely available statistical programming language R [29] in version 3.0.2.

In order to carry out hierarchical clustering and calculate heatmaps source code from the heatmap.2 function, provided as part of the “gplots” CRAN package (version 2.12.1) has been used. In more detail, hierarchical clustering relying on the Euclidian distance has been carried out on quantile normalized data (normalization has been done by the “preprocessCore” package using standard parameters). In order to estimate the linear dependencies between the different replicates the Pearson correlation coefficient has been calculated (standard “stats” package). If not mentioned explicitly, significance vales have been calculated using t-test (unpaired, two-tailed, homoscedastic variance). As alpha level, 0.05 has been used through the manuscript. In order to evaluate whether homoscedastic t-test is applicable to the data, normal distribution was verified using the Shapiro Wilk test and the variance has been tested using Bartletts test (both provided by the R “stats” package.

Finally, functional enrichment analysis has been carried out using TAM, the tool for annotations of microRNAs, which is freely accessible online (http://202.38.126.151/hmdd/tools/tam.html/ [23]). TAM relies on the hypergeometric distribution to calculate p-values.

### Availability of supporting data

The array data have been deposited in the Gene expression Omnibus GEO and are freely accessible under reference GSE56590.
